# Supplementary material for: Spectrally stable thermal emitters enabled by material-based high-impedance surfaces
Source: Nanoscale Adv. 2022 Nov 28;5(3):650–8. doi: 10.1039/d2na00633b (PMC9890674; doi:10.1039/d2na00633b)
Supplement: NA-005-D2NA00633B-s001 [file NA-005-D2NA00633B-s001.pdf]

Electronic Supplementary Material (ESI) for Nanoscale Advances.
This journal is © The Royal Society of Chemistry 2022

Nanoscale

ARTICLE TYPE

Cite this: DOI: 00.0000/xxxxxxxxxx

SUPPLEMENT INFORMATION: Spectrally stable thermal emitters enabled by material-based high-impedance surfaces

David Navajas,[a] José M. Pérez-Escudero,[a] and Iñigo Liberal[a]

Received Date
Accepted Date

DOI: 00.0000/xxxxxxxxxx

Radiative thermal engineering with subwavelength metallic bodies is a key element for heat and energy management applications, communication and sensing. Here, we numerically and experimentally demonstrate metallic thermal emitters with narrowband but extremely stable emission spectra, whose resonant frequency does not shift with changes on the geometry of the system, the angle of observation and/or polarization. Our devices are based on epsilon-near-zero (ENZ) substrates acting as material-based high-impedance substrates. They do not require from complex nanofabrication processes, thus being compatible with large-area and low-cost applications.

1 Salisbury Screen

1.1 Salisbury Screens with a lower dielectric constant

Both the bandwidth and the angular variability of a Salisbury screen electromagnetic absorber are inversely proportional to the permittivity of the spacer, ε_{spa}. For this reason, a germanium (Ge) spacer with a large permittivity in the wavelength range of interest, $\varepsilon_{spa} \simeq 16$, has been selected as a competitive test. In other words, it provides the toughest test for the proposed spectrally stable thermal emitters. We remark that Salisbury screen's with spacers with a lower permittivity would present a worse performance in terms of narrow bandwidth and angular stability. To illustrate this point, Fig. S1 depicts the performance of a Salisbury screen designed with a spacer of relative permittivity $\varepsilon_{spa} = 4$ and thickness of 1.825 μm. As expected, the bandwidth of the absorption band is broader than that of the Ge-based Salisbury screen presented in the main text, and it is also more susceptible to variations in the thickness of the metal layer (see Fig. S1a), and angle changes (see Figs. S1b and S1c)

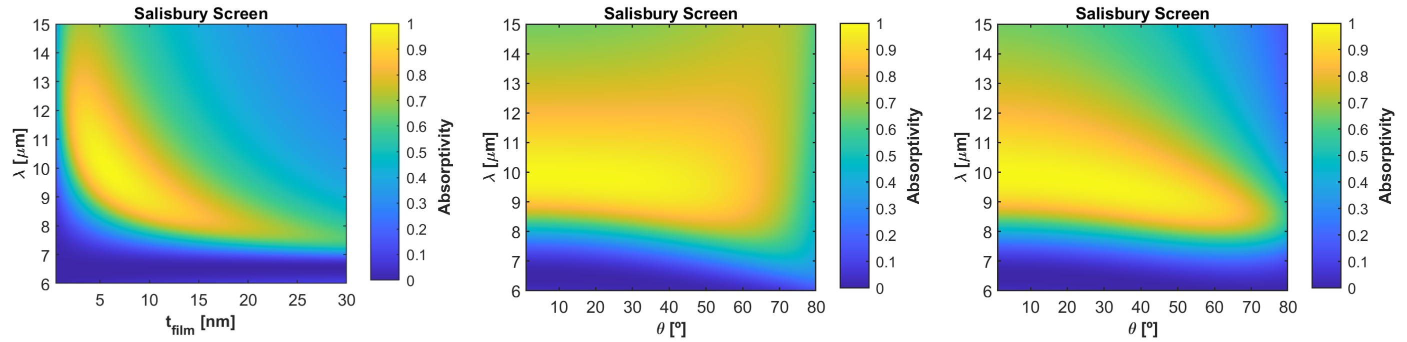

(a) (b) (c)

Fig. S 1 (a) Numerical calculation varying the thickness of the film for a salisbury screen with a spacer of ε_{spa}=4 an a thickness of 1.6 μm. (b) Angle sweep for a selected thickness of 6 nm in transverse magnetic mode. (c) Angle sweep for a selected thickness of 6 nm in transverse electric mode.

1.2 Impact of the metallic film inductance on the resonant frequencies

The results reported in Fig 2 of the main text reveal that the frequency of operation of a Salisbury screen depends of the thickness of the metallic film. Here, we provide an explanation for this effect by using the transmission line model reported in Fig. S2a. In this model, the configurations are calculated as electrical circuits: the metallic film is represented by a shunt impedance, the spacer is represented by a transmission line, and the back mirror is presented as a short-circuit. The input impedance of the full substrate (spacer + mirror) is calculated with the following equation:

[a] *Department of Electrical, Electronic and Communications Engineering, Institute of Smart Cities (ISC), Public University of Navarre (UPNA), 31006 Pamplona, Spain; E-mail: david.navajas@unavarra.es, inigo.liberal@unavarra.es*

$$Z_{sub} = Z_{spa} \frac{Z_L + jZ_{spa} tan(\beta t_{spa})}{Z_{spa} + jZ_L tan(\beta t_{spa})} \quad (1)$$

where Z_{sub} represents the input impedance, the propagation constant is $\beta = \frac{2\pi\sqrt{\varepsilon_{spa}}}{\lambda}$, $Z_{spa} = \frac{1}{\sqrt{\varepsilon_{spa}}}$ is the impedance of the spacer (Ge in our case), Z_L the impedance of the mirror and t_{spa} is the thickness of the spacer. For an ideal back mirror we have $Z_L = 0$ and the inpur impendace reduces to $Z_{sub} = jZ_{spa}tan(\beta t_{spa})$.

For a resistive film, the absorption is maximized at the point where $tan(\beta t_{spa}) \rightarrow \infty$ and the Salisbury screen is a pure $\lambda/4$ resonator. However, for metallic films with an internal inductance the absorption is maximized at a point where a capacitive substrate impedance (represented by a negative value of $tan(\beta t_{spa})$) compensates. For this reason, the peak of absorption is shifted to shorter wavelengths as the film thickness increases, and it asymptotically tend to the $tan(\beta t_{spa}) = 0$ condition for thick films.

In order to further clarify this point, two scenarios have been calculated: one when the the Z_{film} is completely resistive (R_{film}=550 Ω), and the other one when Z_{film} is complex and calculated with the equation shown (See figure 2b). For the simulations performed, the $tan(\beta t_{spa}) \rightarrow \infty$ is satisfied at the wavelenghts of 2.7 μm, 4.32 μm and 10.35 μm. Our results show that absorption peaks of the resistive film exactly match with those wavelengths, while the absorption peaks for the complex film are shifted to shorter wavelengths.

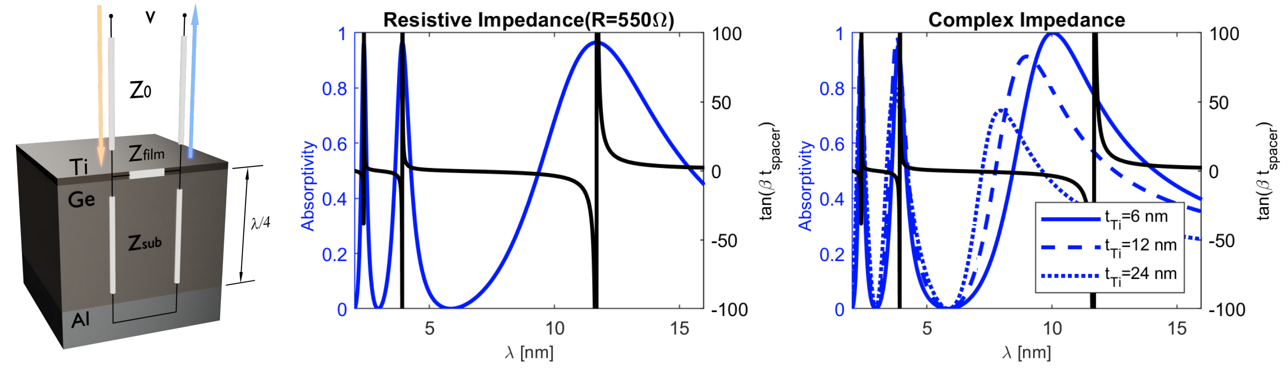

(a) (b) (c)

Fig. S 2 (a) Representation of the transmission line model of the salisbury screen. (b) Numerical calculation of the absorptivity of a resistive Ti film impedance ($R_{film} = 500\Omega$) for thickness of 6 nm. (b) Numerical calculation of the absorptivity of a complex Ti film impedance for thicknesses of 6 nm, 12 nm y 24 nm.

Additional calculations have been made to illustrate the performance of a resistive film (see Fig. S3). It can be concluded from our results that purely resistive films exhibit a better stability both against thickness and angle of incidence, albeit at the cost of a broader bandwidth.

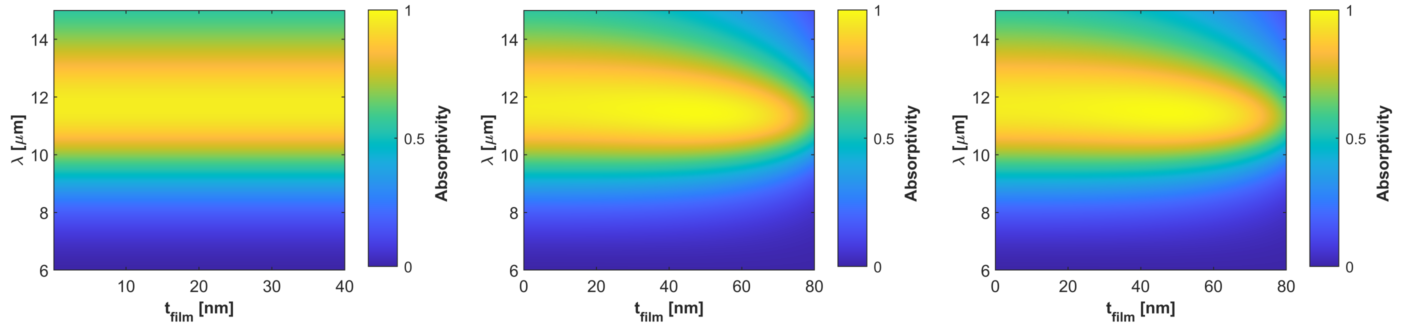

(a) (b) (c)

Fig. S 3 (a) Numerical calculation varying the thickness of a film with a resistive impedance of 550Ω using the transmission line model for a salisbury screen with a spacer of 730 nm. (b) Angle sweep for a selected thickness of 6 nm in transverse magnetic mode. (c) Angle sweep for a selected thickness of 6 nm in transverse electric mode.

2 Silicon Carbide (SiC) substrate

The accuracy of the calculations made with the reflectivity difference in the experimental part is verified in the following study for different thicknesses (see Fig. S4) and angles of incidence (see Fig. S5), in comparison with the absorptivity of the film and the measurements. As it could be observed, in the region of study (Reststrahlen band), the reflectivity difference fits very well with both the measurements and the absorptivitiy of the film, even though it is a pessimistic estimation. Notice that the maximal deviation if observed for the sample with 5 nm thickness, where the roughness is comparable to the thickness of the film.

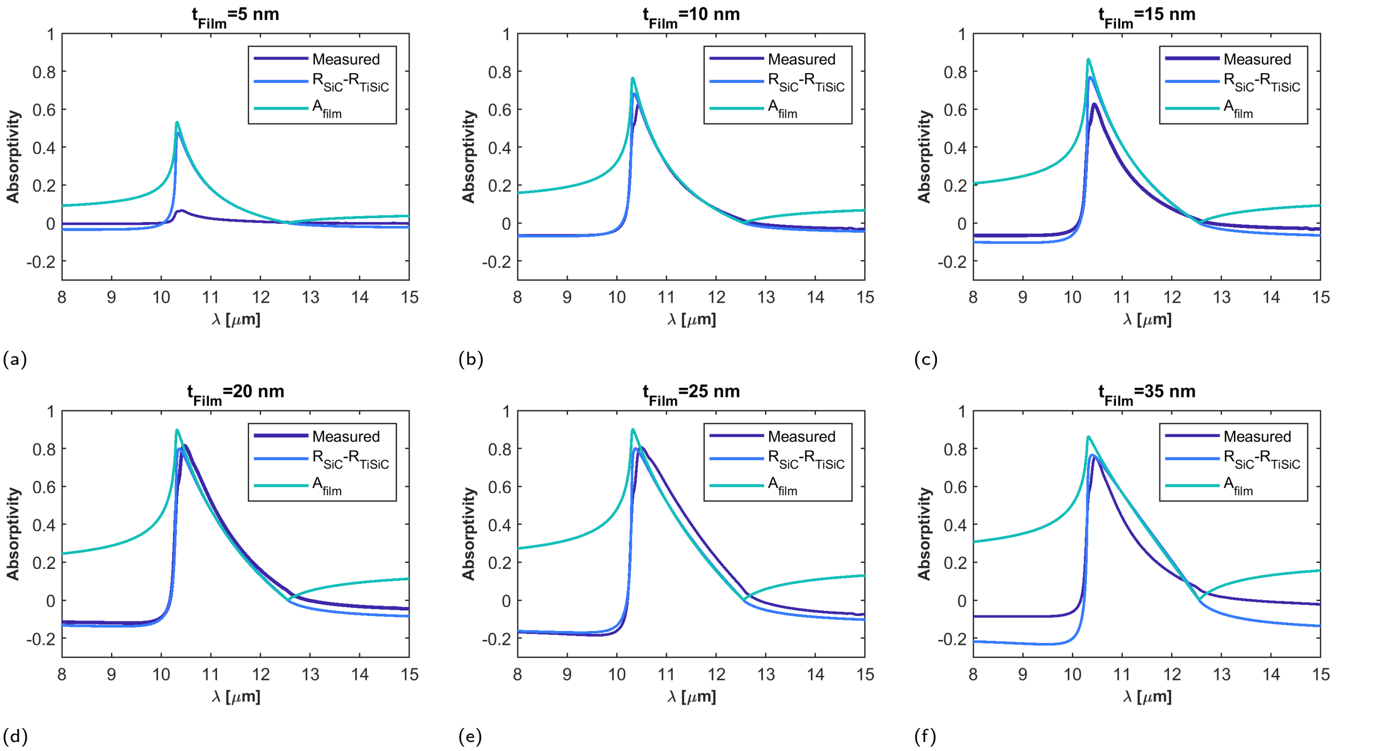

Fig. S 4 Comparison between the measurements, the film absorptivity calculated with the transmission line model, and the reflectivity difference for all of the samples at normal incidence.

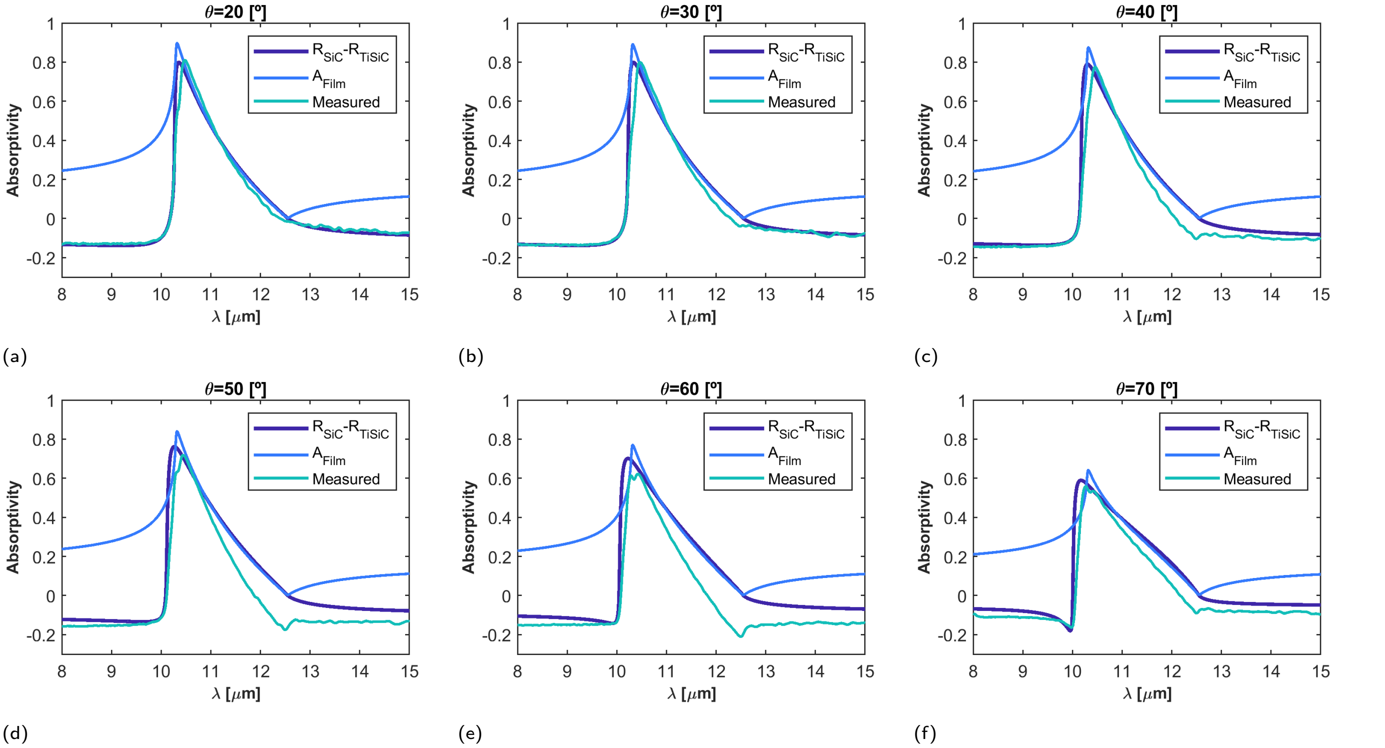

Fig. S 5 Comparison between the measurements, the film absorptivity calculated with the transmission line model and the reflectivity difference for the sample of 20 nm of Ti film thickness for incidences from 20°to 70°.
